# Effects of exposure to low-dose ionizing radiation on changing platelets: a prospective cohort study

**DOI:** 10.1186/s12199-021-00939-z

**Published:** 2021-01-25

**Authors:** Ning Liu, Yang Peng, Xinguang Zhong, Zheng Ma, Suiping He, Ying Li, Wencui Zhang, Zijun Gong, Zhenjiang Yao

**Affiliations:** 1 Department of Epidemiology and Health Statistics, School of Public Health, Guangdong Pharmaceutical University, Guangzhou, 510310 Guangdong China; 2 Cancer Research Centre, Cancer Council Queensland, Fortitude Valley, Brisbane, 4006 Australia; 3 School of Clinical Medicine, The University of Queensland, Herston, 4006 Australia; 4 The Sixth People’s Hospital of Dongguan, Dongguan, 532008 Guangdong China

**Keywords:** Low-dose ionizing radiation, Occupation exposure, Hematological, Platelets

## Abstract

**Background:**

Numerous studies have concentrated on high-dose radiation exposed accidentally or through therapy, and few involve low-dose occupational exposure, to investigate the correlation between low-dose ionizing radiation and changing hematological parameters among medical workers.

**Methods:**

Using a prospective cohort study design, we collected health examination reports and personal dose monitoring data from medical workers and used Poisson regression and restricted cubic spline models to assess the correlation between changing hematological parameters and cumulative radiation dose and determine the dose-response relationship.

**Results:**

We observed that changing platelet of 1265 medical workers followed up was statistically different among the cumulative dose groups (*P* = 0.010). Although the linear trend tested was not statistically significant (*P*_trend_ = 0.258), the non-linear trend tested was statistically significant (*P*_non-linear_ = 0.007). Overall, there was a correlation between changing platelets and cumulative radiation dose (a change of β^a^ 0.008 × 10^9^/L during biennially after adjusting for gender, age at baseline, service at baseline, occupation, medical level, and smoking habits; 95% confidence interval [CI] = 0.003,0.014 × 10^9^/L). Moreover, we also found positive first and then negative dose-response relationships between cumulative radiation dose and changing platelets by restricted cubic spline models, while there were negative patterns of the baseline service not less than 10 years (− 0.015 × 10^9^/L, 95% CI = − 0.024, − 0.007 × 10^9^/L) and radiation nurses(− 0.033 × 10^9^/L, 95% CI = − 0.049, − 0.016 × 10^9^/L).

**Conclusion:**

We concluded that although the exposure dose was below the limit, medical workers exposed to low-dose ionizing radiation for a short period of time might have increased first and then decreased platelets, and there was a dose-response relationship between the cumulative radiation dose and platelets changing.

## Background

Many studies have shown that high-dose ionizing radiation obtained by accidental exposure or radiation therapy can damage human health, such as inducing cancer, invading the hematopoietic system, and causing leukemia [1–5]. However, few studies have documented that low-dose ionizing radiation during occupational exposure has the health effects [6–8].

Outcomes of health examination belonging to the workers in low-dose ionizing radiation have given considerable attention by the public since the second half of the twentieth century. One research in this area has focused on cancer incidence and mortality among workers who are occupationally exposed to low-dose ionizing radiation [9]. In recent years, a study has found that the hematopoietic system of the human body has concerned to be one of the most sensitive biological indicators in radiobiology [10], and the changes of blood cells may lead to diseases including organism immunity to drop, infection, inflammation, anemia, coagulation dysfunction, leukemia, myelodysplastic syndrome, and hemophilic cell syndrome. Leukemia is the most serious blood disease caused by ionizing radiation [11]. Moreover, the correlation between ionizing radiation doses and the leukemia mortality has been reported by many studies [12, 13]. And a model of the hematopoietic system for the Techa River residents chronically exposed to ionizing radiation describes a relative decline in blood cell counts (leukocytes erythrocytes and platelets) caused by the dose rate [14]. However, these researches are limited on ionizing radiation dose and hematopoietic system among medical workers. Therefore, we implemented a study to investigate whether low-dose ionizing radiation exposure is associated with a change in hematological parameters among medical workers who were engaged in radiation occupations, and if so, whether there is a dose-response relationship between the cumulative radiation dose and changing hematological parameters or not.

## Materials and methods

### Study participants

This study prospectively analyzed the health examination and personal dose monitoring data of medical workers who performed occupational health examinations in a chronic disease prevention hospital in Guangdong Province from 2015 to 2019 and had been occupationally exposed to low-dose ionizing radiation for more than 1 year on the study period. The government mandates medical radiation workers conducting biennially occupational health examination including hematological parameters at accrediting institutes.

We collected health examination data; excluded subjects who had a history of hematopoietic system diseases, cancer before the beginning of follow-up, recent infections, taking acetylsalicylic acid or antibiotics, and pregnancy during the study period; and included subjects who were aged ≥ 18 years old at baseline. None of the participants’ doses in the detected radiation exposure exceeded the occupational exposure limit by *International Commission on Radiological Protection* set.

### Assessment of the cumulative radiation dose

According to *Methods of personal dose monitoring for radiation workers*, radiation exposure doses of medical workers, who wear the badge on their chest during work, was monitored by the thermoluminescent dosimeter to record the radiation dose. It was suggested that the annual equivalent dose limit of radiation workers was reduced to an average 20 mSv in 5 years with no more than 50 mSv for 1 year [15].

Due to the loss of several personal doses monitoring data, we used the notional dose, which was the average dose of the same occupation in the same year to supplement the missing data, and calculated the cumulative radiation dose by the job exposure matrix [16, 17]. We divided the subjects into categories based on the inter-quartile range of the cumulative radiation dose. In order to evaluate hematological parameter changing related to covariates, we selected the low-dose group as the reference.

### Assessment of hematological parameters

We collected hematological parameters data by the automatic blood cell analyzer measuring which was part of the health examination for medical workers including hemoglobin, platelets, red blood cells, and white blood cells. The reference range of male hemoglobin was 120–175 g/L and red blood cell was 4.0–5.8 × 10^12^/L, and female was 110–150 g/L and 3.5–5.1 × 10^12^/L, respectively; white blood cell was 4.0–9.5 × 10^9^/L and platelet was 100–350 × 10^9^/L.

### Statistical analysis

The hematological parameters and the personal dose monitoring data were collected from each subject and analyzed to identify changing patterns between hematological parameters and cumulative radiation dose. The characteristics of the study population were described by mean ± SD for quantitative data. Comparison between two groups was carried out by two-sided Student’s *t* test and three and above groups by one-way ANOVA; if the variances are unequal, Welch’s methods (Welch test or Welch’s ANOVA) was used. Poisson regression was used to analyze the associated factors when the trend test was non-linear between the dose group and hematological parameters with the hematological parameters as the dependent variable and continuously changing cumulative radiation dose as the independent variable. To adjust confounding factors, gender, service at baseline, occupation, and medical level were considered to be covariates, accounting for the changes in hematological parameters associated with covariate in the further analysis. And we evaluated the dose-relationship between the cumulative radiation dose and changes in hematological parameters by restricted cubic spline models which had five knots at 1th, 25th, 50th, 75th, and 95th centiles to flexibly model the association [18, 19]. In all analyses, a two-sided significance level of 0.05 was adopted. All statistical analyses were performed using the Stata15.1 software program.

## Results

### Characteristics of the study population

There were 1265 subjects, of the 1285 participants in the study cohort, including 916 men and 349 women. Twenty participants were excluded because they replaced the health examination hospital during the study and lost the data onto health examination reports on the hospital which was our study location. The cumulative radiation dose was 0.200–31.272 mSv, the median value was 3.313 [2.586, 3.754] mSv, and the doses were divided into 0.200–2.586 mSv, 2.586–3.754 mSv, and 3.754–32.00 mSv by the inter-quartile range. The average age at the baseline of medical workers was 37.07 ± 10.03 years (range, 18.54–71.60 years), and the mean servicing was 9.56 ± 8.28 years. The range of male hemoglobin was 93.13–183.50 g/L, red blood cell was 4.14–7.58 × 10^12^/L, white blood cell was 3.10–14.90 × 10^9^/L, and platelet was 112.90–451.30 × 10^9^/L and female was 84.10–154.10 g/L, 3.33–6.54 × 10^12^/L, 2.90–11.80 × 10^9^/L, and 72.90–552.00 × 10^9^/L, respectively.

Table 1 describes the characteristics of the study population. The differences in the distribution of hemoglobin are statistically significant (*P* < 0.05) mainly in that male hemoglobin is lower than female, radiation nurses have the lowest hemoglobin on occupation, smokers have higher hemoglobin than non-smokers, and hemoglobin increases slightly with the baseline service. The differences in the distribution of red blood cells are statistically significant (*P* ≤ 0.001), mainly in that females have lower red blood cells than males, radiation nurses have the lowest red blood cells on occupation, and red blood cells decrease with the baseline age and the effect of smoking is similar to hemoglobin. The differences in the distribution of white blood cells are statistically significant (*P* < 0.05), mainly in that female’s white blood cells are lower than male, white blood cells decrease with baseline age, and the effect of smoking is similar to hemoglobin. The differences in the distribution of platelets are statistically significant (*P* < 0.001) mainly in that males have lower level platelets than females, physicians have lower platelets on occupation, and platelets decrease with baseline age.

**Table 1:** The characteristics of the study population on hematological parameters

Variable	No. (*n* = 1265)	Hemoglobin (g/L)	Red blood cells (10^12^/L)	White blood cells (10^9^/L)	Platelets (10^9^/L)	*F/t*^1^	*F/t*^2^	*F/t*^3^	*F/t*^4^	*P* value^1^	*P* value^2^	*P* value^3^	*P* value^4^
Gender						− 33.10	20.96	3.33	− 6.20	< 0.001	< 0.001	0.001	< 0.001
Male	916 (72.41)	130.91 ± 10.77	5.29 ± 0.51	6.37 ± 1.57	226.23 ± 49.92								
Female	349 (27.59)	153.41 ± 10.82	4.63 ± 0.48	6.04 ± 1.48	246.09 ± 53.56								
Age at baseline						− 0.51	3.39	2.18	3.73	0.613^#^	0.001^#^	0.029	< 0.001
< 40 years	818 (64.66)	147.05 ± 15.22	5.15 ± 0.61	6.35 ± 1.56	235.69 ± 51.23								
≥ 40 years	447 (35.34)	147.48 ± 13.90	5.04 ± 0.53	6.15 ± 1.52	224.41 ± 51.81								
Service at baseline						− 2.11	0.18	− 1.86	0.78	0.035	0.857^#^	0.063	0.433
< 10 years	787 (62.21)	146.52 ± 14.95	5.11 ± 0.61	6.21 ± 1.51	232.60 ± 51.20								
≥ 10 years	478 (37.79)	148.32 ± 14.38	5.11 ± 0.55	6.38 ± 1.60	230.24 ± 52.53								
Occupation						65.33	26.92	2.37	6.82	< 0.001^#^	< 0.001	0.072^#^	< 0.001^#^
Physician	808 (63.87)	149.25 ± 13.37	5.17 ± 0.55	6.23 ± 1.49	227.50 ± 51.19								
Radiation technician	296 (23.40)	147.19 ± 16.04	5.12 ± 0.61	6.49 ± 1.67	240.15 ± 54.73								
Nurse	111 (8.77)	132.78 ± 11.32	4.66 ± 0.59	6.21 ± 1.66	243.15 ± 47.47								
Others	50 (3.95)	146.21 ± 17.28	5.03 ± 0.55	6.05 ± 1.37	224.31 ± 40.28								
Medical level													
Primary	220 (17.39)	147.17 ± 14.29	5.12 ± 0.61	6.25 ± 1.60	229.38 ± 50.90	0.01	0.01	0.05	1.61	0.986	0.987	0.947	0.201
Secondary	406 (32.09)	147.12 ± 14.60	5.11 ± 0.56	6.28 ± 1.53	228.91 ± 52.37								
Third	639 (50.51)	147.27 ± 15.04	5.11 ± 0.59	6.29 ± 1.55	234.28 ± 51.49								
Smoking habits						− 8.35	− 3.75	− 4.94	1.29	< 0.001^#^	< 0.001^#^	< 0.001^#^	0.197
No	941 (74.39)	145.33 ± 14.82	5.08 ± 0.61	6.14 ± 1.48	232.81 ± 51.87								
Yes	324 (25.61)	152.65 ± 13.17	5.20 ± 0.49	6.66 ± 1.68	228.51 ± 51.14								
The cumulative radiation dose (mSv)						0.09	0.81	2.38	4.68	0.407	0.444	0.094^#^	0.010^#^
0~2.586		148.13 ± 14.53	5.14 ± 0.57	6.13 ± 1.42	225.33 ± 47.35								
2.586~3.757		146.97 ± 14.93	5.11 ± 0.59	6.35 ± 1.64	235.75 ± 55.15								
3.758~31.272		146.70 ± 14.67	5.08 ± 0.59	6.28 ± 1.48	230.38 ± 48.31								

^#^The variance is unequal, and Welch’s ANOVA was used

^1^The characteristics of the study population on hemoglobin

^2^The characteristics of the study population on red blood cells

^3^The characteristics of the study population on white blood cells

^4^The characteristics of the study population on platelets

### Effect of cumulative radiation dose and hematology parameters among medical radiation workers

There was a statistically significant difference in platelets among the cumulative radiation dose group (*P* = 0.010) which the 2.586~3.757-mSv group had the highest level, 235.75 ± 55.15 (10^9^/L). Although there was no statistical significance on the linear trend test of their association (*P*_trend_ = 0.258), the non-linear trend test was statistically significant (*P*_non-linear_ = 0.007). And there were no statistical differences to the cumulative radiation dose groups and other hematological parameters such as hemoglobin, red blood cells, and white blood cells (*P* > 0.05) (Table 1). In the following analysis, a hierarchical Poisson regression analysis should be conducted based on gender, age at baseline, service at baseline, occupation, medical level, and smoking habits.

### Correlation between platelets changing and cumulative radiation dose

Overall, there was a correlation between platelets changing and the cumulative radiation dose (*β*^a^ = 0.008 × 10^9^/L, 95% CI = 0.003, 0.014 × 10^9^/L) after adjusting to gender, services at baseline, occupation, medical level, and smoking. Platelets in male showed an increase trend with the cumulative radiation dose (*β*^a^ = 0.013 × 10^9^/L, 95% CI = 0.007, 0.019 × 10^9^/L), while females had little correlation (*β*^a^ = − 0.002 × 10^9^/L, 95% CI = − 0.012, 0.008 × 10^9^/L). The platelets increased in baseline age not less than 40 years older (*β*^a^ = 0.020 × 10^9^/L, 95% CI = 0.010, 0.029 × 10^9^/L), and the baseline age less than 40 also showed an increase (*β* = 0.009 × 10^9^/L, 95% CI = 0.002, 0.015 × 10^9^/L), but there was no statistical difference in this association (*P* = 0.262) after adjusting relation factors. The platelet increased to baseline service less than 10 years (*β*^a^ = 0.021 × 10^9^/L, 95% CI = 0.014, 0.027 × 10^9^/L), while the opposite trend showed at baseline service for 10 years or more (*β*^a^ = − 0.015 × 10^9^/L, 95% CI = − 0.024, − 0.007 × 10^9^/L). Radiation technicians platelets increased (*β*^a^ = 0.045 × 10^9^/L, 95% CI = 0.033, 0.056 × 10^9^/L), radiation nurses decreased (*β*^a^ = − 0.033 × 10^9^/L, 95% CI = − 0.049, − 0.016 × 10^9^ /L). There was an increase in secondary medical level (*β*^a^ = 0.019 × 10^9^/L, 95% CI = 0.010, 0.028 × 10^9^/L), so did smoking (*β*^a^ = 0.037 × 10^9^/L, 95 % CI = 0.026, 0.047 × 10^9^/L). Among the characteristics of females, baseline age less than 40 years old, radiation physician, tertiary medical level, and non-smoking, there was no statistically significant association between cumulative radiation dose and platelets (*P* > 0.05). The multivariate Poisson regression analysis of platelet changing into medical radiation workers was shown in Table 2.

**Table 2:** Multivariate Poisson regression analyses for the biennially changes in platelets

Variable	*β* (95% CI)	*P*	*β*^a^(95% CI)	*P*^a^
All	0.011 (0.006, 0.016)	< 0.001	0.008 (0.003, 0.014)	0.001
Gender				
Male	0.012 (0.006, 0.018)	< 0.001	0.013 (0.007, 0.019)	< 0.001
Female	− 0.001 (− 0.010, 0.010)	0.955	− 0.002 (− 0.012, 0.008)	0.751
Age at baseline				
< 40 years	0.009 (0.002, 0.015)	0.007	0.004 (− 0.003, 0.010)	0.262
≥ 40 years	0.023 (0.014,0.032)	< 0.001	0.020 (0.010, 0.029)	< 0.001
Service at baseline				
< 10 years	0.027 (0.020, 0.033)	< 0.001	0.021 (0.014, 0.027)	< 0.001
≥ 10 years	− 0.014 (− 0.023, − 0.006)	0.001	− 0.015 (− 0.024, − 0.007)	< 0.001
Occupation				
Physician	− 0.002 (− 0.008, 0.004)	0.530	− 0.002 (− 0.009, 0.004)	0.497
Radiation technician	0.047 (0.036, 0.059)	< 0.001	0.045 (0.033, 0.056)	< 0.001
Nurse	− 0.031 (− 0.048, − 0.015)	< 0.001	− 0.033 (− 0.049, − 0.016)	< 0.001
Others	0.043 (0.016, 0.069)	0.002	0.037 (0.008, 0.066)	0.012
Medical level				
Primary	0.006 (− 0.007, 0.020)	0.336	0.015 (0.002, 0.029)	0.027
Secondary	0.017 (0.007, 0.026)	< 0.001	0.019 (0.010, 0.028)	< 0.001
Third	0.010 (0.003, 0.017)	0.004	0.004 (− 0.003, 0.011)	0.274
Smoking habits				
No	0.007 (0.001, 0.013)	0.020	− 0.001 (− 0.007, 0.005)	0.838
Yes	0.025 (0.015, 0.035)	< 0.001	0.037 (0.026, 0.047)	< 0.001

^a^Estimate based on Poisson regression model, adjusted for gender, age at baseline, service at baseline, occupation, medical level, and smoking habits

**Fig. 1:**
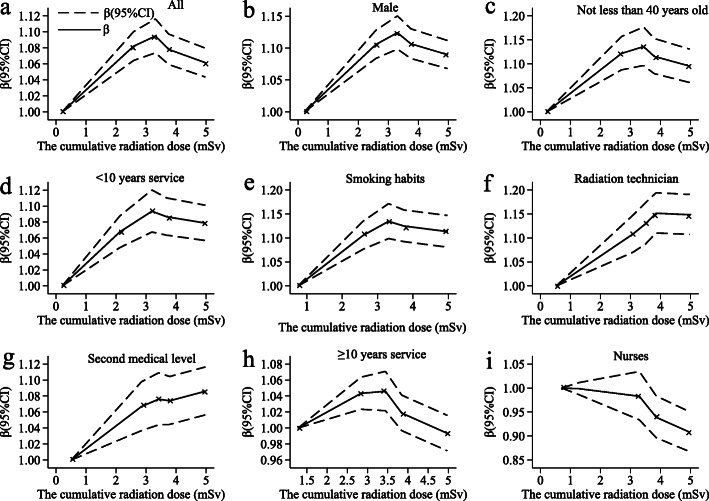
The dose-response relationship between platelets changing and the cumulative radiation dose. We recorded the changes of platelets in **a** the whole population, **b** male, **c** not less than 40 years old, **d** less than 10 years of service, **e** smoking habits, **f** radiation technician, **g** second medical level, **h** over 10 years of service, and **i** nurses occupations. The solid and dashed lines are *β* values and corresponding 95% confidence intervals. *β* coefficients were estimated using Poisson regression

The analysis of the restricted cubic spline model (Fig. 1a ) shows that the platelets have a non-linear dose-response relationship of cumulative radiation dose after adjusting to gender, baseline age, baseline service, occupation, medical level, and smoking. The median cumulative radiation dose is 3.313 mSv, and the doses are within 3.313 mSv showing a positive dose-response relationship; greater than 3.313 mSv showing a negative dose-response relationship (*P*_non-linear_ = 0.007). Overall, the cumulative radiation dose has a ⋀-shaped association with the platelet level; the estimated platelet level coefficient β per 1 mSv increase in the cumulative radiation dose is 0.031 (0.024–0.038) below 3.313 mSv and − 0.004 (− 0.007 to − 0.001) above this point. The platelet trend of males, baseline age not less than 40 years older, baseline service less than 10 years, and smoking participants is basically similar to the overall trend (Fig. 1b–e), but there are slightly different to radiation technicians and secondary medical level (Fig. 1f, g), which the basic trend is consistent with the overall situation. However, the platelet shows a negative dose-response relationship of baseline service not less than 10 years and radiation nurse (Fig. 1h, i).

## Discussion

This study found that low-dose ionizing radiation is related to changing platelets, and there is dose-response evidence for a change of platelets among medical workers exposed to occupational ionizing radiation in hospital. This report updates the effect of low-dose ionizing radiation on medical workers’ hematological parameters and explores the dose-response relationship between the both.

### Overall associations of low-dose ionizing radiation and changing platelets among medical workers

As shown in Table 2, occupational exposure to ionizing radiation has an impact on the hematological parameters of medical workers; what is more obvious is the effect on platelets in the short term. The platelet count of males increases in the cumulative radiation doses after low-dose ionizing radiation, whereas the opposite is no significant change in females; this reflects that the gender may be a confounding factor of cumulative radiation dose and platelets’ changing into hematological parameters. The increase in platelets not less than 40 years old suggests that the risk of thrombosis in middle-aged radiation workers is increased. When service at the baseline was less than 10 years, platelets of the participant showed an increasing trend with the cumulative radiation dose, whereas there was an opposite trend in changing platelets if the service was greater than 10 years; this means that service at the baseline (< 10 years) has an impact on promoting platelets after irradiation, whereas inhibiting platelets may cause coagulation disorders and affect bone marrow hematopoietic function after 10 years servicing, and there may be a healthy worker effect. The table also shows that the platelet changes of radiation technician and nurses have opposite trends as the cumulative radiation dose increases, indicating that they have the potential capability to influence changing from hematological parameters in different occupations. Only the second medical level increased platelets in the study population of the cumulative radiation dose, which also showed that different medical levels had an impact on changing the hematological parameters of the medical workers. This may be caused by the different protective measures and education levels of different. By the way, the primary medical level has a statistical significance of multivariate analysis; we consider that there is an interaction between factors after adjusting the related factors.

### The dose-response relationship between changing of platelets and cumulative radiation dose

Although Fig. 1a shows a dose-response relationship between the cumulative radiation dose and changing platelets by restricted cubic spline models, the shape of dose-response curve is non-linearity. On the whole, the platelet levels of medical workers related to the cumulative radiation dose and the multivariate analysis also supported the relationship. In general, the changing in platelets increases first and then decreases non-linearly as the cumulative radiation dose after adjusting to confound factors such as gender, age at the baseline, service at the baseline, occupation, medical level, and smoking. However, the changing trend of platelet counts for baseline service not less than 10 years, and radiation nurses are contrary to the overall. This may be related to the fact that there are more female nurses. Radiation professional women have more or less menstrual problems due to work stress, and menorrhagia is the main manifestation which is consistent with the physical condition caused by thrombocytopenia. The service period of more than 10 years indicates that the body’s compensatory response mechanism may be impaired, causing radiation damage to the body and leading to thrombocytopenia. Whether the changing of platelet level is increased or decreased to the cumulative radiation dose, it shows that low-dose ionizing radiation has a certain impact on the hematological parameter changing of medical workers, thereby affecting health. Therefore, the findings of the epidemiological study of changing into hematological parameters among medical workers with the low-dose ionizing radiation are confirmed by our study, which consists of biennial health examinations and personal dose monitoring since 2015.

### Mechanisms of changing in hematological parameters

The hematopoietic system of the human body that is sensitive to ionizing radiation has been evaluated in many studies [10, 20, 21]. According to reports, ionizing radiation exposure causes biological effects on human health including hematological parameters that change sooner or later, and hematopoietic syndrome is observed in animals and humans after systemic radiation exposure [22], including myelodysplastic disease by platelet elevation causing and chronic myelogenous leukemia [23–25]. The most important cause of hematopoietic syndrome is that induction to apoptosis in hematopoietic stem cells and hematopoietic progenitor cells which is primarily responsible for ionizing radiation inducing acute bone marrow injury, depending on ionizing radiation doses [23, 26]. The findings provide a reasonably consistent picture of changing into hematological parameters associated with exposure to radiation. Moreover, there is a supplement to the risk assessment of low-dose ionizing radiation based on animal models [20, 27, 28].

### Comparison with other studies

Despite the limited epidemiological and experimental data, several studies had shown the effects which were low-dose ionizing radiation on hematopoietic system, and early manifestations of changing in hematological parameters existed possibility. Long-term low-dose ionizing radiation can cause acute and chronic reactions against the hematopoietic system, just as radiation-related leukemia occurs mainly due to radiation-induced DNA damage [11]. A study on chronically irradiated residents of Techa riverside villages found an influence on the hematopoietic system which was the correlation between chronic exposure dose rates and granulocytes, platelets, and red blood cells by modeling analysis [29]. As we all know, changing into the levels of platelets, white blood cells, and red blood cells are important signs of leukemia. From occupational leukemia studies, the cumulative radiation dose bellowing the effective limit is associated with leukemia incidence or mortality among the radiation-monitored workers [12, 13, 30, 31]. Wan-Ling Hsu also found that there is a non-linear dose-response relationship between radiation dose and leukemia, and the excess risk will continue until 55 years after irradiation, especially acute myeloid leukemia [24]. In addition, some experiments, which were associated with hematopoietic stem cells’ radio-sensitivity and implemented by 90Sr ionizing radiation on rats’ bone marrow and mesenchymal stem cells, also demonstrated the effects of ionizing radiation on the hematopoietic system [32, 33]. Significant associations with radiation for the hematological parameters were reported on medical occupational workers at a large hospital with a lower mean of the total white blood cell and the neutrophils than the control group [34], while these studies may have limitations on sample selection risk factors of changing into hematological parameter levels and personal dose monitoring. The low-dose mouse model experiment also found ionizing radiation damaged to the hematopoietic system including granulocytes in the irradiated mice increased by 1.7 times of the seventh day of the experiment [20]. Another study also found this effect of cumulative dose, long-term ionizing radiation inhibits blood cell production, and only a few past years of exposure contribute to the observed hematological parameter declines to include platelets [14, 35]. XiangHong Li also mentioned the effect of low- and medium-dose gamma radiation on the hematopoietic system basing on CD2F1 mice that the blood cell count decreased with whole body irradiation after 3 and 5 Gy keeping below the baseline for 28–42 days, while the lymphocytes and monocytes increased after 0.5 Gy peaking at day 3 to day 14, and mouse bone marrow (BM) progenitor cells were significantly suppressed on the first day after 0.5–5 Gy lasting low levels up to 42 days [27]. Therefore, low-dose ionizing radiation damages the hematopoietic system changing hematological parameters, which is consistent with the results of this study. It is worth mentioning that we also found that the platelets may increase as the ionizing radiation dose in the early. The result should arouse the attention on researchers in the biological effects of radiation damage early.

The relationship between low-dose ionizing radiation dose and hematological parameters is unclear. A study found that, although most of the parameters were below the normal range and were disturbed in the majority of the radiation-exposed workers, these associations were weak between annual average effective dose (0.29–1.91 mSv) and hematological parameters, comparing with blood parameters with the normal range of radiation-exposed workers and radiation-unexposed workers [36]. Another study on expansive epidemiologic entitled the Million Person Study of Low-Dose Radiation Health Effects found that medical workers had been detected any late-occurring health effects of radiation exposures experienced gradually in time including hematological parameter changing [37]. Moreover, there have been few researches on the dose-response relationship between low-dose ionizing radiation and hematological parameters among medical workers, and the most informative low-dose radiation studies to date provide little evidence for a relationship between mortality from non-malignant diseases and radiation dose [38]. When ionizing radiation dose less than 0.5 Gy, epidemiology cannot prove that the risk increases on health damage [7]. As Alexander Vaiserman [9] mentioned, the biological effects of low-dose ionizing radiation still have many areas worthy of further study. This study proves the correlation between low-dose ionizing radiation and platelets, which can provide a basis of hematological parameters as indicators on biological effects, so as to avoid serious, irreversible, and irreparable radiation damage.

With the exception of the study of all Korean radiation workers [39], most studies did not adjust confounding factors of potential lifestyle or other, and some of them did not have or lacked some personal dose monitoring data. Consequently, the statistical power of most low-dose studies was limited, existed some potential biases, with increasing possibility results of false positive and false negative. Although the health effects of low-dose ionizing radiation (< 100 mSv) are still controversial, human epidemiological and clinical studies have shown that the influencing factors of low-dose ionizing radiation and health effects are socio-demographic, genetics, radiation composition, and source, lifestyle, and other environments exposure [40]. Therefore, this study found that platelet levels of medical workers increased to the cumulative dose after adjusting to gender, age at baseline, service at the baseline, occupation, and medical level.

### Strengths and limitations

This study has several strengths. It not only has a professional population, but also has a large sample size. There is a minimal bias, medical surveillance bias, because all are eligible for services of free special medical. And there is a blind method which many people know nothing about the dose they received. Strict quality controls ensured the association with cumulative radiation dose with the hematological parameters. The experimental operations from blood collection of testing are performed strictly by laboratory-rich physicians in order to avoid experimental errors including random errors and gross errors. In addition, the personal dose monitoring data is more complete and accurate, and special confounding factors are recorded which is including lifestyle and socio-demographic.

This study also has several limitations. First, individual dose monitoring data is missing for a few cases. Although the nominal dose has been used to fill in this study, the radiation dose estimated by the job exposure matrix may have measurement bias, and medical workers should be urged to wear the thermoluminescent dosimeter to complete personal dose monitoring data. Second, the analysis of confounding factors is incomplete, which is lacking information about drugs, socioeconomic status, exercise, and other environmental exposure, resulting in underestimated or overestimated relevance. Third, the lag effects because of early occupational exposure changing hematological parameters may occur, while these effects are very small. Finally, there are healthy worker effects on occupational studies, and some statistically significant findings occur accidentally due to short follow-up period. Although the theory of blood parameters as indicators of radiation damage biological effects is not systematic and mature, our findings lay the foundation for this theory.

## Conclusions

The unique feature of this study is that there is a positive first and then negative dose-response relationship between the cumulative radiation dose and hematological parameter changing. Even though all dose data are below the detection limit, this result still provides the strongest evidence that low-dose ionizing radiation exposure is associated with platelet counts among medical workers. Therefore, platelets may be a sensitive biomarker of low-dose ionizing radiation, and relevant measures may be taken before the symptoms of the hematopoietic system appearing by detecting hematological parameters to assess the early effects of ionizing radiation exposure.

## Data Availability

The datasets generated and/or analyzed during the current study are not publicly available due to the contract with chronic disease prevention hospital regarding information protection but are available from the corresponding author on reasonable request.

## References

[1] Behjati S, Gundem G, Wedge DC, Roberts ND, Tarpey PS, Cooke SL (2016). Mutational signatures of ionizing radiation in second malignancies. Nat Commun..

[2] Grant EJ, Cologne JB, Sharp GB, Eguchi H, Stevens RG, Izumi S (2018). Bioavailable serum estradiol may alter radiation risk of postmenopausal breast cancer: a nested case-control study. Int J Radiat Biol..

[3] Hong JY, Han K, Jung JH, Kim JS (2019). Association of exposure to diagnostic low-dose ionizing radiation with risk of cancer among youths in South Korea. JAMA Netw Open..

[4] Richardson DB, Cardis E, Daniels RD, Gillies M, Haylock R, Leuraud K (2018). Site-specific solid cancer mortality after exposure to ionizing radiation: a cohort study of workers (INWORKS). Epidemiology..

[5] Seo S, Lee D, Seong KM, Park S, Kim SG, Won JU, Jin YW (2018). Radiation-related occupational cancer and its recognition criteria in South Korea. Ann Occup Environ Med..

[6] Ahmad IM, Abdalla MY, Moore TA, Bartenhagen L, Case AJ, Zimmerman MC. Healthcare workers occupationally exposed to ionizing radiation exhibit altered levels of inflammatory cytokines and redox parameters. *Antioxidants (Basel).* 2019;8(1). doi:10.3390/antiox8010012. 10.3390/antiox8010012 PMC6356728 30609664

[7] Baselet B, Rombouts C, Benotmane AM, Baatout S, Aerts A (2016). Cardiovascular diseases related to ionizing radiation: the risk of low-dose exposure (Review). Int J Mol Med..

[8] McLean AR, Adlen EK, Cardis E, Elliott A, Goodhead DT, Harms-Ringdahl M, et al. A restatement of the natural science evidence base concerning the health effects of low-level ionizing radiation. *Proc Biol Sci.* 2017;284(1862). doi:10.1098/rspb.2017.1070. 10.1098/rspb.2017.1070 PMC5597830 28904138

[9] Vaiserman A, Koliada A, Zabuga O, Socol Y (2018). Health impacts of low-dose ionizing radiation: current scientific debates and regulatory issues. Dose Response..

[10] Waselenko JK, MacVittie TJ, Blakely WF, Pesik N, Wiley AL, Dickerson WE (2004). Medical management of the acute radiation syndrome: recommendations of the Strategic National Stockpile Radiation Working Group. Ann Intern Med..

[11] Fleenor CJ, Higa K, Weil MM, DeGregori J (2015). Evolved cellular mechanisms to respond to genotoxic insults: implications for radiation-induced hematologic malignancies. Radiat Res..

[12] Little MP, Wakeford R, Borrego D, French B, Zablotska LB, Adams MJ (2018). Leukaemia and myeloid malignancy among people exposed to low doses (<100 mSv) of ionising radiation during childhood: a pooled analysis of nine historical cohort studies. The Lancet Haematology..

[13] Leuraud K, Richardson DB, Cardis E, Daniels RD, Gillies M, O’Hagan JA (2015). Ionising radiation and risk of death from leukaemia and lymphoma in radiation-monitored workers (INWORKS): an international cohort study. The Lancet Haematology..

[14] Akushevich IV, Veremeyeva GA, Dimov GP, Ukraintseva SV, Arbeev KG, Akleyev AV, Yashin AI (2010). Modeling deterministic effects in hematopoietic system caused by chronic exposure to ionizing radiation in large human cohorts. Health Phys..

[15] Publication ICRP (2007). 105. Radiation protection in medicine. Ann ICRP..

[16] Choi S, Kang D, Park D, Lee H, Choi B (2017). Developing asbestos job exposure matrix using occupation and industry specific exposure data (1984-2008) in Republic of Korea. Saf Health Work..

[17] Ilar A, Klareskog L, Saevarsdottir S, Wiebert P, Askling J, Gustavsson P, Alfredsson L (2019). Occupational exposure to asbestos and silica and risk of developing rheumatoid arthritis: findings from a Swedish population-based case-control study. RMD Open..

[18] Bhaskaran K, Dos-Santos-Silva I, Leon DA, Douglas IJ, Smeeth L. Association of BMI with overall and cause-specific mortality: a population-based cohort study of 3·6 million adults in the UK. *Lancet Diabetes Endocrinol*. 2018 Dec;6(12):944-953. doi: 10.1016/S2213-8587(18)30288-2. Epub 2018 Oct 30. PMID: 30389323; PMCID: PMC6249991. 10.1016/S2213-8587(18)30288-2 PMC6249991 30389323

[19] Lee DH, Keum N, Hu FB, Orav EJ, Rimm EB, Willett WC, Giovannucci EL. Predicted lean body mass, fat mass, and all cause and cause specific mortality in men: prospective US cohort study. *BMJ*. 2018 Jul 3;362:k2575. doi: 10.1136/bmj.k2575. PMID: 29970408; PMCID: PMC6028901. 10.1136/bmj.k2575 PMC6028901 29970408

[20] Hoehn D, Pujol-Canadell M, Young EF, Serban G, Shuryak I, Maerki J (2019). Effects of high- and low-LET radiation on human hematopoietic system reconstituted in immunodeficient mice. Radiat Res..

[21] Shao L, Luo Y, Zhou D (2014). Hematopoietic stem cell injury induced by ionizing radiation. Antioxid Redox Signal..

[22] Billings PC, Romero-Weaver AL, Kennedy AR (2014). Effect of gender on the radiation sensitivity of murine blood cells. Gravit Space Res..

[23] Gault N, Verbiest T, Badie C, Romeo PH, Bouffler S (2019). Hematopoietic stem and progenitor cell responses to low radiation doses - implications for leukemia risk. Int J Radiat Biol..

[24] Hsu WL, Preston DL, Soda M, Sugiyama H, Funamoto S, Kodama K (2013). The incidence of leukemia lymphoma and multiple myeloma among atomic bomb survivors: 1950-2001. Radiat Res..

[25] Radivoyevitch T, Sachs RK, Gale RP, Molenaar RJ, Brenner DJ, Hill BT (2016). Defining AML and MDS second cancer risk dynamics after diagnoses of first cancers treated or not with radiation. Leukemia..

[26] Fliedner TM, Graessle DH, Meineke V, Feinendegen LE (2012). Hemopoietic response to low dose-rates of ionizing radiation shows stem cell tolerance and adaptation. Dose Response..

[27] Li X, Cui W, Hull L, Smith JT, Kiang JG, Xiao M (2018). Effects of low-to-moderate doses of gamma radiation on mouse hematopoietic system. Radiat Res..

[28] Wang C, Nakamura S, Oshima M, Mochizuki-Kashio M, Nakajima-Takagi Y, Osawa M (2015). Compromised hematopoiesis and increased DNA damage following non-lethal ionizing radiation of a human hematopoietic system reconstituted in immunodeficient mice. Int J Radiat Biol..

[29] Smirnova OA, Akleyev AV, Dimov GP (2014). Analysis of hematopoiesis dynamics in residents of Techa riverside villages chronically exposed to nonuniform radiation: modeling approach. Health Phys..

[30] Jung J, Choi HR, Cho BS, Park S, Myong JP, Kang MY, Kim HJ. Establishment and operation of a cooperative program to identify work-related acute myeloid leukemia in a general hospital. *Ann Occup Environ Med.* 2019;31:e33. doi:10.35371/aoem.2019.31.e33. 10.35371/aoem.2019.31.e33 PMC6941933 31915524

[31] Park C, Choi S, Kim D, Park J, Lee S (2014). A case of chronic myeloid leukemia in a diagnostic radiographer. Ann Occup Environ Med..

[32] Russu IZ, Rodionova NK, Bilko DI, Bilko NM (2015). Pattern changes in quantitative and qualitative markers of hematopoietic stem cells during acute and chronic exposure to 90Sr isotope in cell culture. Probl Radiac Med Radiobiol..

[33] Russu IZ, Rodionova NK, Bilko DI, Bilko NM (2017). Mesenchymal stem and progenitor cells of rats' bone marrow under chronic action of ionizing radiation. Probl Radiac Med Radiobiol..

[34] Caciari T, Capozzella A, Tomei F, Nieto HA, Gioffrè PA, Valentini V (2012). Professional exposure to ionizing radiations in health workers and white blood cells. Ann Ig..

[35] Akushevich IV, Veremeyeva GA, Dimov GP, Ukraintseva SV, Arbeev KG, Akleyev AV, Yashin AI (2011). Modeling hematopoietic system response caused by chronic exposure to ionizing radiation. Radiat Environ Biophys..

[36] Shahid S, Mahmood N, Chauhdry MN, Sheikh S, Ahmad N (2014). Assessment of impacts of hematological parameters of chroning radiation exposed workers in hospitals. FUUAST J BIOL..

[37] Boice JD Jr, Cohen SS, Mumma MT, Ellis ED. The Million Person Study whence it came and why. *Int J Radiat Biol.* 2019:1–14. 10.1080/09553002.2019.1589015. 10.1080/09553002.2019.1589015 30831042

[38] Vrijheid M, Cardis E, Ashmore P, Auvinen A, Bae JM, Engels H (2007). Mortality from diseases other than cancer following low doses of ionizing radiation: results from the 15-Country Study of nuclear industry workers. Int J Epidemiol..

[39] Seo S, Lim WY, Lee DN, Kim JU, Cha ES, Bang YJ (2018). Assessing the health effects associated with occupational radiation exposure in Korean radiation workers: protocol for a prospective cohort study. BMJ Open..

[40] Tang FR, Loganovsky K (2018). Low dose or low dose rate ionizing radiation-induced health effect in the human. J Environ Radioact..

